# Podiatrists’ perspectives of the provision of foot health education for people with Rheumatoid Arthritis

**DOI:** 10.1186/1757-1146-3-S1-O11

**Published:** 2010-12-20

**Authors:** Andrea Graham, Alison Hammond, Anita Williams

**Affiliations:** 1 University of Salford, School of Health, Sports and Rehabilitation Sciences, Salford, UK

## Introduction

Patient education is considered to be a key role for podiatrists in the management of patients with Rheumatoid Arthritis (RA). However there is no evidence that identifies or supports the most appropriate strategies and content for its delivery. The aim of this study was to identify the nature and content of the current provision by podiatrists in relation to foot health education for people with RA. Any potential barriers to its provision were explored.

## Method

Twelve members (all female) of the Northwest Podiatry Clinical Effectiveness Group for Rheumatology volunteered to take part in a Focus Group, ranging from newly qualified podiatrists with an interest in working within rheumatology to those with extensive years of experience managing patients with RA (working within Primary and Secondary care). The dialogue was recorded digitally, transcribed verbatim and analysed using a structured, thematic approach by the lead author. The full transcription was verified as accurate by the focus group to ensure trustworthiness of the data.

## Results

Five overarching themes emerged from the data, each with it’s own set of sub-themes, these were, 1. The Essence of Patient Education [information provision & empowerment,] 2. The content [what, why, when & by whom], 3. Barriers to Provision [external, psychosocial, educational, concordance & professional experience], 4. The therapeutic relationship [patient/practitioner knowledge & attitudes, influence of age & gender, role/title confusion, taboo subject areas] and 5. Tools of the trade [group v individual, verbal & written, audio-visual, web-based].

## Discussion

This methodological approach of this study has revealed aspects of patient education, which this group of podiatrists find to be most influential in its overall delivery from their perspective. This adds new knowledge as no previous study has been carried out. The lead author is currently exploring the same area with patient participants. Ultimately the aim is to achieve the development of a patient-centred and negotiated approach to the provision of foot health education for people with RA.

